# Supplementary material for: Asymmetric Synthesis of Quaternary Hydantoins via a Palladium-Catalyzed Aza-Heck Cyclization
Source: J Am Chem Soc. 2025 Nov 14;147(49):44692–8. doi: 10.1021/jacs.5c16022 (PMC12703750; doi:10.1021/jacs.5c16022)
Supplement: Supplementary file 2 [file ja5c16022_si_002.zip › All NMR FID Files/S8 conformer 2/S8-B_ProtonNMR/1/pdata/1/email_EM06-036-isomer#2-dry-1H_1_1.pdf]

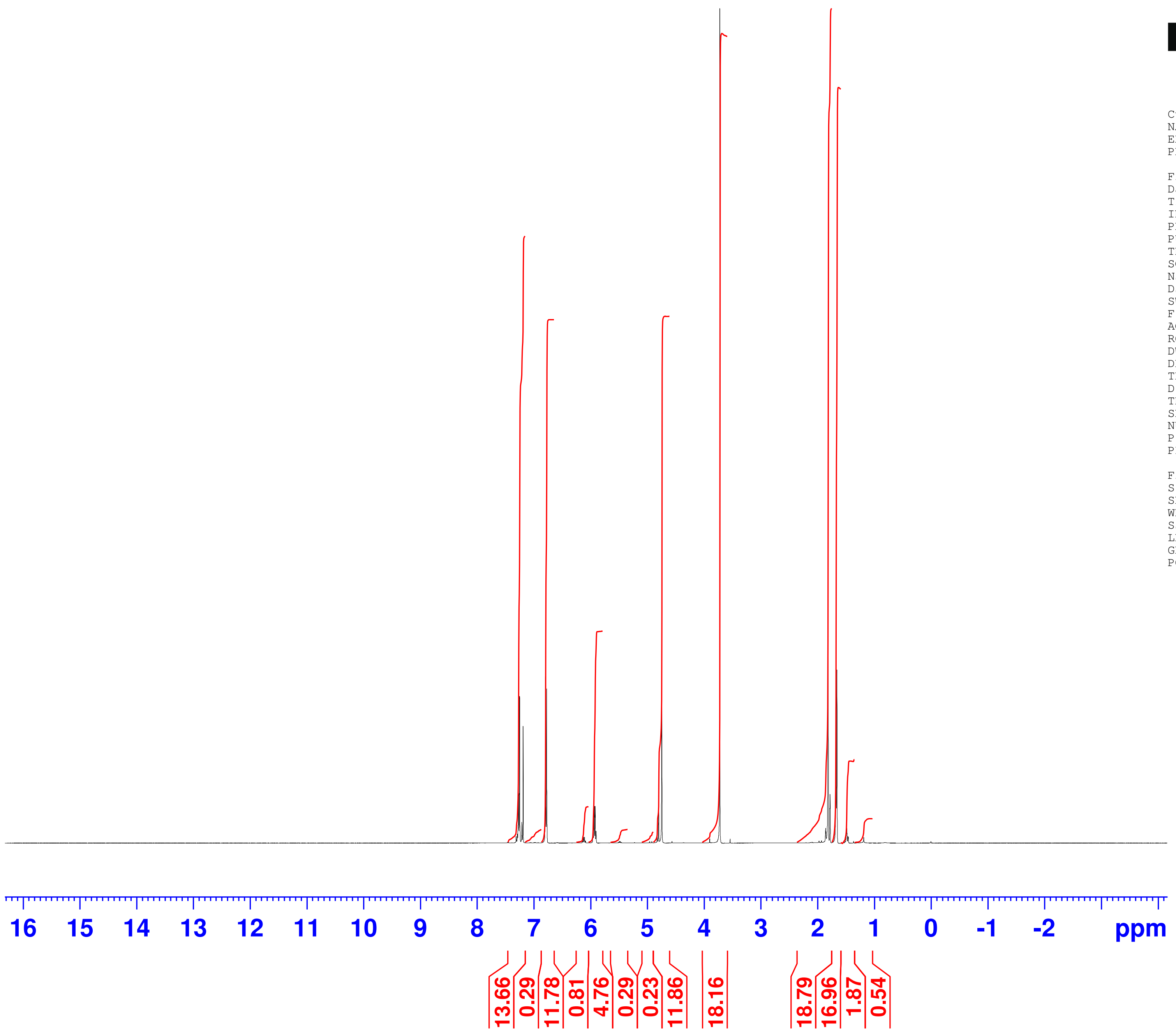

BRUKER

```
Current Data Parameters
NAME      EM06-036-isomer#2-dry-1H
EXPNO                    1
PROCNO                   1

F2 - Acquisition Parameters
Date_             20250211
Time                  8.39 h
INSTRUM   Avance Neo 400 U of DE Brown Lab
PROBHD    Z104450_0412 (
PULPROG                 zg
TD                   65536
SOLVENT              CDCl3
NS                      32
DS                       2
SWH               8196.721 Hz
FIDRES            0.250144 Hz
AQ               3.9976959 sec
RG                     101
DW                  61.000 usec
DE                   13.33 usec
TE                   298.0 K
D1             10.00000000 sec
TD0                      1
SFO1           400.1324708 MHz
NUC1                    1H
P1                   15.00 usec
PLW1            8.31639957 W

F2 - Processing parameters
SI                   65536
SF             400.1300382 MHz
WDW                     EM
SSB                      0
LB                    0.30 Hz
GB                       0
PC                    1.00
```
